# Identification and Expression of Neurotrophin-6 in the Brain of *Nothobranchius furzeri*: One More Piece in Neurotrophin Research

**DOI:** 10.3390/jcm8050595

**Published:** 2019-04-30

**Authors:** Adele Leggieri, Chiara Attanasio, Antonio Palladino, Alessandro Cellerino, Carla Lucini, Marina Paolucci, Eva Terzibasi Tozzini, Paolo de Girolamo, Livia D’Angelo

**Affiliations:** 1 Department of Veterinary Medicine and Animal Productions, University of Naples Federico II, I-80137 Naples, Italy; adele.leggieri@unina.it (A.L.); chiara.attanasio@unina.it (C.A.); lucini@unina.it (C.L.); degirola@unina.it (P.d.G.); 2 Interdepartmental Center for Research in Biomaterials (CRIB) University of Naples Federico II, I-80125 Naples, Italy; a.palladino1986@gmail.com; 3 Center for Advanced Biomaterials for Healthcare-Istituto Italiano di Tecnologia, I-80125 Naples, Italy; 4 Laboratory of Biology Bio@SNS, Scuola Normale Superiore, I-56126 Pisa, Italy; alessandro.cellerino@sns.it (A.C.); eva.terzibasi@sns.it (E.T.T.); 5 Laboratory of Biology of Aging, Leibniz Institute on Aging-Fritz Lipmann Institute, D-0445 Jena, Germany; 6 Department of Sciences and Technologies, University of Sannio, I-82100 Benevento, Italy; paolucci@unisannio.it; 7 Department of Biology and Evolution of Marine Organisms, Stazione Zoologica Anton Dohrn, I-80121 Naples, Italy

**Keywords:** neurotrophin-6, phylogeny, LNA probe, riboprobe, neuroanatomy, fish, aging

## Abstract

Neurotrophins contribute to the complexity of vertebrate nervous system, being involved in cognition and memory. Abnormalities associated with neurotrophin synthesis may lead to neuropathies, neurodegenerative disorders and age-associated cognitive decline. The genome of teleost fishes contains homologs of some mammalian neurotrophins as well as a gene coding for an additional neurotrophin (NT-6). In this study, we characterized this specific neurotrophin in the short-lived fish *Nothobranchius furzeri*, a relatively new model for aging studies. Thus, we report herein for the first time the age-related expression of a neurotrophin in a non-mammalian vertebrate. Interestingly, we found comparable expression levels of NT-6 in the brain of both young and old animals. More in detail, we used a locked nucleic acid probe and a riboprobe to investigate the neuroanatomical distribution of NT-6 mRNA revealing a significant expression of the neurotrophin in neurons of the forebrain (olfactory bulbs, dorsal and ventral telencephalon, and several diencephalic nuclei), midbrain (optic tectum, longitudinal tori, and semicircular tori), and hindbrain (valvula and body of cerebellum, reticular formation and octavolateral area of medulla oblongata). By combining in situ hybridization and immunohistochemistry, we showed that NT-6 mRNA is synthesized in mature neurons. These results contribute to better understanding the evolutionary history of neurotrophins in vertebrates, and their role in the adult brain.

## 1. Introduction

*Nothobranchius furzeri* is a novel model organism for aging research [1,2,3] being its captive lifespan the shortest ever recorded for a vertebrate [4]. The life cycle of *N. furzeri*, indeed, is characterized by explosive growth [5] and rapid expression of aging phenotypes at behavioral, histological, and molecular levels [6,7,8]. Concerning the brain, *N. furzeri* displays typical aging hallmarks, including lipofuscin accumulation, age-dependent gliosis and rapid decay of adult neurogenesis [9]. The identification of specific genes under positive selection revealed potential candidates to explain the compressed lifespan of this fish. Several age-related genes, indeed, are under positive selection in *N. furzeri* and long-lived species, including humans, raising the intriguing hypothesis that the same gene could underlie evolution of both compressed and extended lifespan [10]. Remarkably, one of the variants in this fish granulin (W449 in the shorter-lived strain and C449 in the longer-lived strain) is within a motif that plays a key role in protein folding, and is mutated in human frontotemporal dementia [11]. The fish variant is predicted to generate functional consequences and is also found in wild fish, thus excluding its derivation from a spurious mutation arisen in the laboratory or from the bottleneck of a rare allele [10].

The assessment of the neurotrophin family came after the identification of the first two members: the nerve growth factor (NGF) [12] and the brain derived neurotrophic factor (BDNF) [13]. These members share stretches of highly homologous amino acid sequences [13], and both support the survival of cultured dorsal root ganglia neurons [14]. Afterwards, three more neurotrophins have been identified in vertebrate genomes: neurotrophin 3 (NT-3), neurotrophin 4 (NT-4) and the fast evolving neurotrophin-5 (NT-5) [15]. Neurotrophins are produced as pre-pro-peptides and undergo proteolytic cleavage before being secreted [16]. They exert many biological effects by their high affinity binding h to the specific tropomyosin-related kinase (Trk) or by a lower affinity interaction with the receptor p75NTR [17]. Specifically, NGF binds to TrkA, NT-3 to TrkC and, with lower affinity, to TrkA, while BDNF and NT-4 bind to TrkB [18]. In addition, p75 receptor can bind to unprocessed or mature neurotrophin and act as co-receptor of Trks [17]. In general, neurotrophins play a role in distinct, as well as partially overlapping, subsets of peripheral and central neurons. Further, individual neurons may also be responsive to more than one neurotrophin at a given time or at subsequent times during development [19]. According to the differential expression and cellular localization of their receptors, neurotrophins can elicit diverse cellular functions in different types of neurons and at different cellular loci [17,18]. Abnormalities associated with neurotrophins synthesis have been linked with neuropathies and neurodegenerative disorders, as well as age-associated cognitive decline.

The genome of teleost fishes contains homologs of the mammalian neurotrophins NGF, BDNF, NT-3 and NT-4 [20] but also a gene coding for one additional neurotrophin originally isolated and cloned in platyfish [21]: neurotrophin-6 (NT-6). The ortholog of this neurotrophic factor in *Danio rerio* [22] and *Cyprinus carpio* [23] was later described as neurotrophin-7 (NT-7). From the biochemical standpoint, NT-6 is featured by the presence of a 22 amino acid residue inserted between the second and third conserved cysteine containing domain. NT-6 promoted the survival of chick sympathetic and sensory dorsal root ganglion neurons, to the same extent of NGF, despite a lower specific activity [21]. Further molecular and phylogenetic studies have provided evidence that, in teleost fishes, NGF and NT-6 are paralogs and originated from duplication of an ancestral gene as consequence of the whole-genome duplication of teleost fishes [24].

Very few studies have been devoted to the role of this neurotrophin in fish, as well as to its expression and morphological distribution. Götz and coworkers [21] documented that NT-6 transcripts are significantly expressed during the embryonic development and adulthood of *Xiphophorus*, in brain, gill, liver and eye while a weak expression is displayed in heart, skin, spleen and skeletal muscles [21]. A very recent paper described NT-6 mRNA during zebrafish embryogenesis (from 12–96 h post fertilization) by whole-mount in situ hybridization [25]. Nittoli et al. reported that early transcript was detected at 16 hpf in two clusters of cells adjacent to the anterior and posterior of the inner ear primordium, and that its expression was lost from 48 h post fertilization onward [25].

In the present study, we investigated the age-related expression of NT-6 in the short-lived teleost, *N. furzeri*. Our findings contribute to: (i) better understand the evolutionary history of neurotrophins in vertebrates; (ii) elucidate their role in vertebrate brain; (iii) demonstrate that NT-6 is expressed in mature neurons of the adult brain; and (iv) document the stable age-associated changes of NT-6 over time.

## 2. Experimental Section

### 2.1. Protocols

The protocols for animal care and use were approved by the appropriate Committee at the University of Naples Federico II (2015/0023947). All animal experimental procedures were carried out in accordance with The European Parliament and The Council of The European Union Directive of 22nd of September 2010 (2010/63/UE) and Italian Law (D.lgs 26/2014).

### 2.2. Animals and Tissue Preparation

Animals, belonging to the long-lived strain MZM 04/10 were used at the following time points: 5 weeks post hatching (wph) (young-adult, age of the sexual maturity) and 27 wph (old animals). Animal maintenance was performed as previously described [26]. To avoid effects of circadian rhythms and feeding, animals were euthanized at 10:00 in a fasted state, with an overdose of anesthetics. They were placed for approximately 5–10 min in a solution containing 1 mg/mL in buffered ethyl 3-aminobenzoate methanesulfonate without prior sedation and observed until no vital signs (body and operculum movement, righting reflex) were observed.

For RNA extraction, 5 fish for each time point (5 and 27 wph) were decapitated, brains were rapidly dissected, kept in sterile tubes (Eppendorf BioPhotometer, Hamburg, Germany) with 500 µL of RNAlater (Qiagen, Hilden, Germany), and stored at 4 °C until the RNA extraction. For fluorescence in situ hybridization (FISH), 5 adult fish (at 20 wph) were decapitated, brains were rapidly excised and fixed in 4% paraformaldehyde (PFA)/PBS overnight (ON) at 4 °C. Then, brains were incubated in 20% sucrose solution ON at 4 °C and successively in 30% sucrose solution ON at 4 °C. Brains were then embedded in cryomount and frozen at −80 °C. Serial transverse and sagittal sections of 12 µm thickness were cut with a cryostat (Leica, Deerfield, IL, USA).

### 2.3. RNA Isolation and cDNA Synthesis

Tissues were taken out of RNAlater and cleaned with sterile pipettes. *N. furzeri* (NFu) total RNA was isolated from 10 animals with QIAzol (Qiagen), according to a modified manufacturer’s protocol [27]. Homogenization was performed using a TissueLyzer II (Qiagen) at 20 Hz for 2–3 × 1 min. Total RNA was then quantized with Eppendorf BioPhotometer. Then, 500 ng of each sample were retrotranscribed to cDNA in a 20 µL volume, using the QuantiTect^®^ Reverse Transcription Kit (Qiagen), following the supplier’s protocol. Newly synthetized cDNAs were then diluted to a final volume of 200 µL with ultra-pure sterile water to an approximate final cDNA’s concentration of 40 ng/µL.

### 2.4. Phylogenetics Analysis

Orthologs of Nfu NT-6 were recovered from Genbank by querying Genbank translated nucleotide sequences with the translated cDNA of NfuNT-6. *D. rerio* glial derived neurotrophic factor (GDNF) sequence was selected as outgroup. All phylogenetic analyses were performed using MEGA X [28]. The analysis involved 17 amino acid sequences of different fish species (differently named as neurotrophin-6/7-like and nerve growth factor-like) and *Homo sapiens* and *Mus musculus*. The most appropriate amino acid substitution model was selected based on Akaike Information Criterion (AIC). Phylogenetic tree was reconstructed by maximum likelihood analysis using a partial deletion (80%) setting, JTT with gamma function and invariant sites as substitution model, and bootstrap analysis.

### 2.5. Quantitative Real Time-PCR

NfuNT6 primers were designed with Primer3 tool [29]. According to the sequence information, one set of primers was designed to quantize NfuNT-6 cDNA: left 5′-GCATTCGTTGAAGTCTGGCT-3′; right 5′-ATCAGGAAGAGCAGGACCAG-3′. Reactions were performed in 20 µL volume containing 1 µL of diluted cDNA, using BrightGreen 2× qPCR MasterMix kit (abm^®^, Richmond, VA, Canada) following the manufacturer’s instructions. Reactions were performed in triplicate and negative control (water) was always included.

### 2.6. Statistical Analysis

Expression levels of NfuNT-6 mRNA were analyzed by the ΔΔCt method and normalized to the housekeeping gene TATA box binding protein (*TBP*): left 5′-CGGTTGGAGGGTTTAGTCCT-3′; right 5′-GCAAGACGATTCTGGGTTTG-3′). Fold changes represent the difference in expression levels between young and old age NfuNT-6 cDNAs, respectively, with young and old age TATA-binding protein (TBP) cDNAs. The relative ΔΔ curve threshold was built on fold changes values and *p*-value was <0.01.

### 2.7. Probe Design

For the neuroanatomical distribution of NfuNT-6 mRNA, two different DIG-labelled probes were employed: a locked nucleic acid (LNA) probe, and an RNA probe (riboprobe). The LNA probe, unlike the RNA probe, contains an extra bridge which connects the 2′ oxygen and 4′ carbon locking the ribose in the 3′ endo conformation. This conformation significantly increases hybridization properties of the probe.

### 2.8. Riboprobe Synthesis

mRNA probes to identify NfuNT-6 mRNA were synthetized by in vitro transcription (IVT) using MAXIscript™ SP6/T7 in vitro transcription kit (Invitrogen by Thermo Fisher Scientific–Catalogue number AM1312, Carlsbad, CA, USA) and following the manufacturer’s instructions. 1 µg of DNA template was transcribed to RNA in 20 µL volume reaction, using NfuNT6 primer associated with the T7 promoter sequence (left 5′-TGGTCCTGCTCTTCCTGATC-3′; T7 right 5′-GGTAATACGACTCACTATAGG_GTGTGTTTGAAGCTGCTCGA-3′) and a DIG RNA Labeling Mix, 10× conc (Roche, Basel, Switzerland) containing digoxigenin labeled uracil. After IVT reaction, product was briefly centrifuged and incubated at 37 °C for 1 h. Then, 1 µL of turbo DNase 1 was added, sample was mixed well and incubated for 15 min at 37 °C. 1 µL of EDTA 0.5 M was added to stop the reaction. Reaction product was analyzed by gel electrophoresis and quantized.

### 2.9. LNA Probe Synthesis

LNA modified DNA oligonucleotide probe, containing an LNA nucleotide at every third position, and labeled at the 59 end only, or at the 59 and 39 ends, with DIG, were supplied by Exiqon Inc. (Vedbaek, Denmark). NT6 probe was designed using the Primer3 primer design program [28] and checked using the LNA Oligo Optimizer tool on the Exiqon website (www.exiqon.com) (see Table 1). Probe sequence was screened against all known *N. furzeri* sequences using BLAST. LNA probe typically shows single nucleotide specificity [30]. Negative control was mismatch probe, designed and synthesized by Exiqon Inc. (see Table 1).

### 2.10. Fluorescence In Situ Hybridization

FISH experiments were performed on cryostat sections using sterile solutions and materials. Diethylpyrocarbonate (DEPC) was added to phosphate-buffered saline (PBS) and water 1 mL/L to inactivate RNase enzymes; solutions were shaken vigorously and autoclaved.

Sections were dried for 2 h at room temperature (RT), well washed in 1× DEPC/PBS and treated with 10 µg/µL Proteinase K (Sigma-Aldrich, St. Louis, MO, USA) 1:200 in DEPC/PBS for 10 min. Proteinase K action was then inactivated by two washes in 2 mg/mL glycine, 5 min each. Sections were post fixed in 4% PFA for 20 min and well washed in 1× DEPC/PBS at RT. Thereafter, the prehybridization was carried out in a hybridization solution (HB) containing 50% formamide, 25% 20× SSC, 50 µg/mL Heparin, 10 µg/mL yeast RNA, 0.1% Tween 20, and 0.92% citric acid at 55 °C (riboprobes) and 42 °C (LNA probes) for 1 h. All probes were denatured for 10 min at 80 °C and sections were then incubated, in HB containing riboprobes concentration of 500 pg/µL, ON at 55 °C and LNA probes concentration of 2 ng/µg, ON at 42 °C. Post-hybridization washes were carried out at 55 °C as follows: 2 × 20 min in 1× SSC, 2 × 10 min in 0.5× SSC, and then in 1× DEPC/PBS at RT. Sections were blocked in blocking solution (BS) containing 10% normal sheep serum heat inactivated and 0.5% blocking reagent (Roche, Hamburg, Germany) for 1 h at RT. After, sections were incubated in a 1:2000 dilution of anti-digoxigenin Fab fragments conjugated with alkaline phosphatase (Roche) in BS, 2 h at RT. Sections were well washed in 1× DEPC/PBS. The chromogenic reaction was carried out by using Fast Red tablets (Sigma-Aldrich) in Tris buffer and incubating the slides at RT in the dark and were observed every 20 min until the signal detection (1–10 h depending on the probe used). After the signal was developed, sections were washed in 1× DEPC/PBS at RT and mounted with Fluoreshield Mounting Medium with DAPI as counterstaining for the nuclei.

### 2.11. Combined In Situ Hybridization and Immunohistochemistry

After the detection of the FISH chromogenic reaction, sections were well washed in DEPC/PBS and incubated at RT for 1 h with blocking serum (normal goat serum 1:5 in PBS containing 0.1% Triton X-100, Sigma) and subsequently with primary antiserum ON at 4 °C. Primary antisera employed were: rabbit polyclonal anti-S100 (1:200, Agilent Dako, Santa Clara, CA, USA, Ref. Z 0311); mouse IgG2b, biotin-XX conjugate anti-HuC/D (1:50, Invitrogen by Thermo Fisher Scientific, Carlsbad, CA, USA, Ref. A21272); mouse monoclonal anti-MAP-2 (1:50, Santa Cruz Biotechnology, Ref. Sc-74422, Dallas, TX, USA), rabbit polyclonal anti-Parvalbumin (1:100 Anti-Parvalbumin Rabbit pAB PC255L-100UL, EMD Millipore, Burlington, MA, USA). DEPC/PBS washes preceded the incubation with the secondary antibodies: goat anti-rabbit IgG (H+L) Alexa fluor™ Plus 488 (1:1000, Invitrogen by Thermo Fisher Scientific, Ref. A32731, Carlsbad, CA, USA) for anti-S100β, anti-Parvalbumin; Alexa Fluor^®^ 488 Streptavidin conjugated (1:800, Jackson Immuno Research Labs, West Grove, PA, USA, Ref. 016540084) for HuC/D; goat anti-mouse IgG (H+L) Alexa fluor™ Plus 488 (1:1000, Invitrogen by Thermo Fisher Scientific, Carlsbad, CA, USA, Ref. A32723) for MAP-2.

### 2.12. Microscopy

FISH images were analyzed with a Zeiss AxioScope AX 1.0 microscope (Carl Zeiss, Jena, Germany) with AxioCam MC5 and AxioVision software. Combined FISH/Immunohistochemistry images were analyzed by Leica–DM6B (Leica, Wetzlar, Germany) and processed with LasX software. The digital raw images were optimized for image resolution, contrast, evenness of illumination, and background using Adobe Photoshop CC 2018 (Adobe Systems, San Jose, CA, USA). Anatomical structures were identified according to the adult *N. furzeri* brain atlas [31].

## 3. Results

### 3.1. Molecular Characterization of NfuNT-6

**Figure 1:**
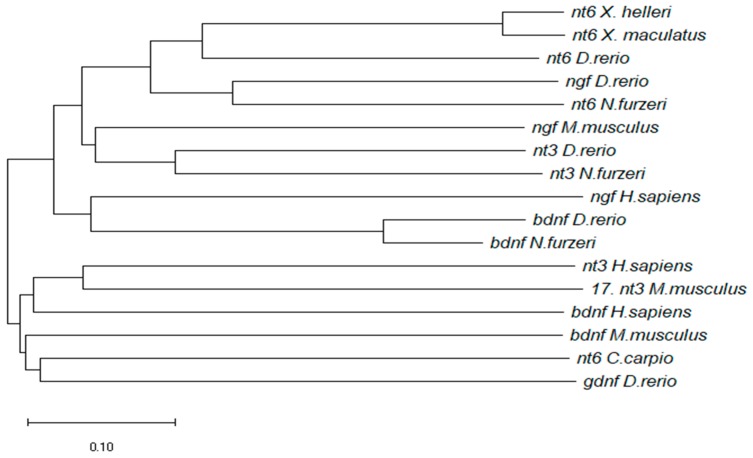
Evolutionary relationships of taxa. The evolutionary history was inferred using the Minimum Evolution method. The optimal tree with the sum of branch length = 4.92138622 is shown. The tree is drawn to scale, with branch lengths in the same units as those of the evolutionary distances used to infer the phylogenetic tree. This analysis involved 17 nucleotide sequences. Codon positions included were 1st+2nd+3rd+Noncoding. All ambiguous positions were removed for each sequence pair (pairwise deletion option). There were 3752 positions in the final dataset.

A putative NT-6 coding sequence was retrieved from the *N. furzeri* transcriptome browser [32]: the sequence is deposited under the Genebank accession number GAIB01193979.1. NfuNT-6 was aligned with the predicted NT-6/7 sequences available in some actinopterygians species (*Xiphophorus*, *Cyprinus carpio* and *Danio rerio*), as well as mammalian and *D. rerio* neurotrophins. NT-4/5 of *D. rerio* was not included in the alignment because it is not still annotated on GenBank. GDNF of *D. rerio* was used as outgroup. The evolutionary history was inferred using the Minimum Evolution method, having selected long nucleotides sequences. The evolutionary distances were computed using the *p*-distance method and are in the units of the number of base differences per site. The analysis involved 17 nucleotides sequences (Figure 1). The ME tree was searched using the Close-Neighbor-Interchange (CNI) algorithm at a search level of 1. The Neighbor-joining algorithm was used to generate the initial tree. All positions containing gaps and missing data were eliminated. The resulting phylogram clearly shows that the *N. furzeri* sequence is nested within a clade of Actinopterygian sequences, and high percentage of conservation with fish neurotrophin-3.

### 3.2. Expression Studies

#### 3.2.1. NfuNT-6 mRNA Expression in Young versus Old Animals

**Figure 2:**
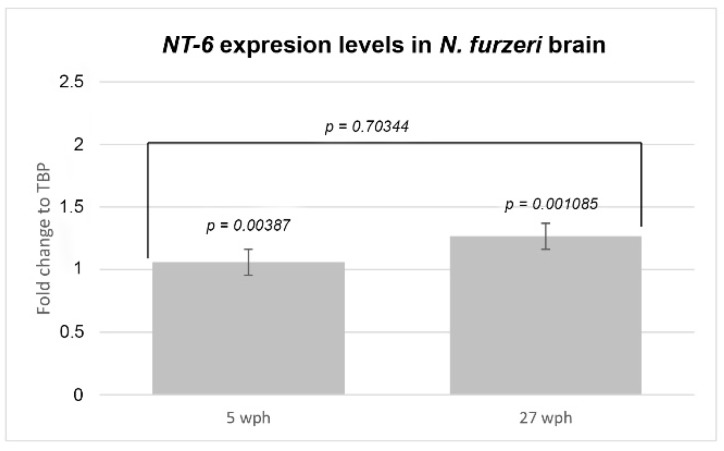
Expression levels of NfuNT-6 mRNA in the brain of young and old *N. furzeri*. Comparable expression levels of NfuNT-6 mRNA in the whole brain of young and old animals (*p*-value: 0.70344). For 5 and 27 wph, NfuNT-6 mRNA was normalized to TBP and expression levels were compared using ΔΔCT method (*p*-value_5wph_ = 0.00387; *p*-value_27wph_ = 0.001085).

We analyzed NfuNT-6 mRNA levels, by qPCR analysis, in brain homogenates of 5 and 27 wph animals. Comparable levels of NfuNT-6 mRNA were found in the brains of young and old animals (*p*-value: 0.70344) (Figure 2). For each time point, NfuNT-6 mRNA was normalized to the reference gene (TBP) and expression levels were compared using the relative delta curve threshold (ΔΔCT) method (*p*-value_5wph_ = 0.00387; *p*-value_27wph_ = 0.001085).

#### 3.2.2. Neuroanatomical Expression of NT-6 mRNA

**Figure 3:**
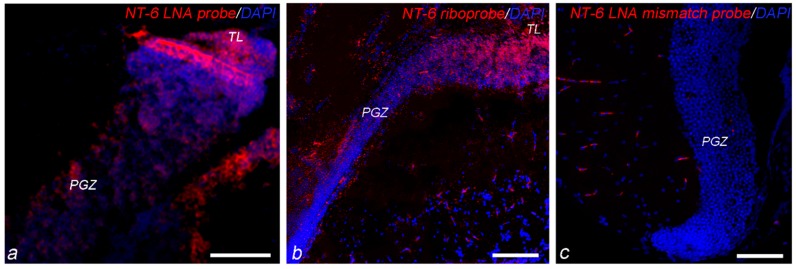
Comparison of riboprobe and LNA and negative control: (**a**) LNA probe and (**b**) Riboprobe to detect NT-6 mRNA expression (red) in the OT, at the margin with TL; and (**c**) mismatch LNA probe in the OT not revealing staining. Scale bar: (**a**,**c**) 25 μm; and (**b**) 12 μm. Abbreviations: PGZ, periventricular grey zone.

LNA and riboprobes (Figure 3a,b) were used to localize the expression of NT-6 mRNA and revealed overlapping distribution patterns. No specific hybridization signal was observed in sections hybridized with the mismatch probe (Figure 3c). Due to overlapping pattern of expression of riboprobe and LNA, the results refer to NT-6 mRNA. The nomenclature follows the *N. furzeri* brain atlas [30]. Recognition of labeled neurons and/or glial cells was based on morphological criteria and by means of different markers: S100β [9,33], HuC/HuD [34] and MAP2 [35]. Purkinje neurons were identified by using parvalbumin as marker.

#### 3.2.3. NT-6 mRNA Expression in Mature Neurons

**Figure 4:**
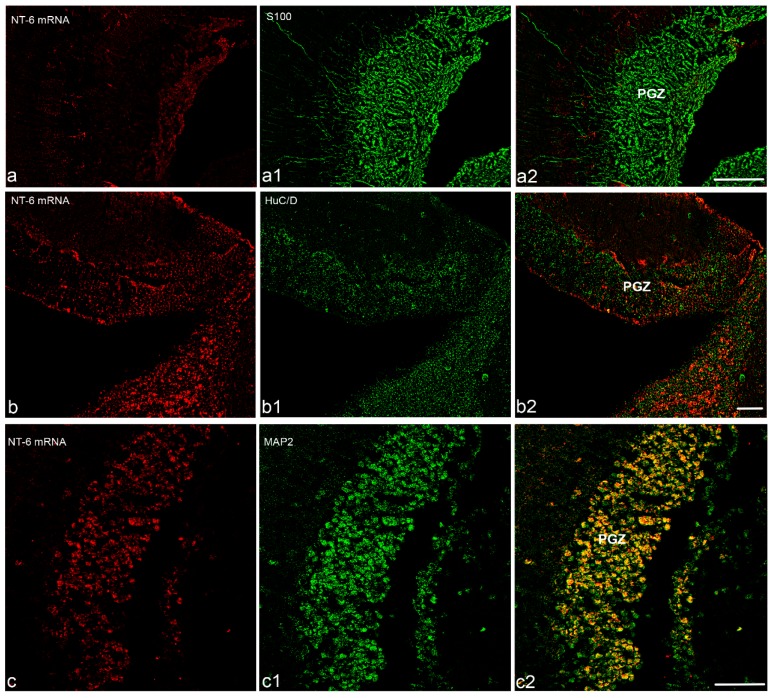
Immunohistochemical characterization of NT-6 mRNA expressing cells in transverse section of optic tectum: (**a**,**a1**,**a2**) single images and merge of NT-6 mRNA and S100β positive cells, showing any co-staining in the rostral part of optic tectum; (**b**,**b1**,**b2**) single images and merge of NT-6 mRNA and HuC/HuD showing any co-staining in the rostral part of optic tectum; and (**c**,**c1**,**c2**) single images and merge of NT-6 mRNA and MAP2 showing that NT-6 is expressed in numerous MAP2 immunopositive. Scale bar: (**a**,**c**) 50 μm; and (**b**) 25 μm. Abbreviations: PGZ, periventricular grey zone.

Before analyzing the pattern of expression of NT-6 over different brain areas, we combined in situ hybridization with immunohistochemistry to identify the phenotype of NT-6 mRNA expressing cells. We conducted the experiment on serial sections of the optic tectum, and we employed markers to identify glial and neuronal populations. S100β was used as marker of glial cells [9,33], HuC/HuD as marker of early-differentiated neurons [34,35], and MAP2 as marker of mature neurons [36,37]. NT-6 mRNA was not expressed in S100β immunoreactive cells (Figure 4a,a1,a2) or in HuC/HuD cells (Figure 4b,b1,b2). NT-6 mRNA signal probe was observed in MAP2 immunoreactive cells in the most posterior part of periventricular grey zone of the optic tectum and in some scarce cells in the most superficial layers of the same brain area (Figure 4c,c1,c2).

#### 3.2.4. Neuroanatomical Distribution of NfuNT-6 mRNA in the Adult *N. furzeri*

##### Forebrain

**Figure 5:**
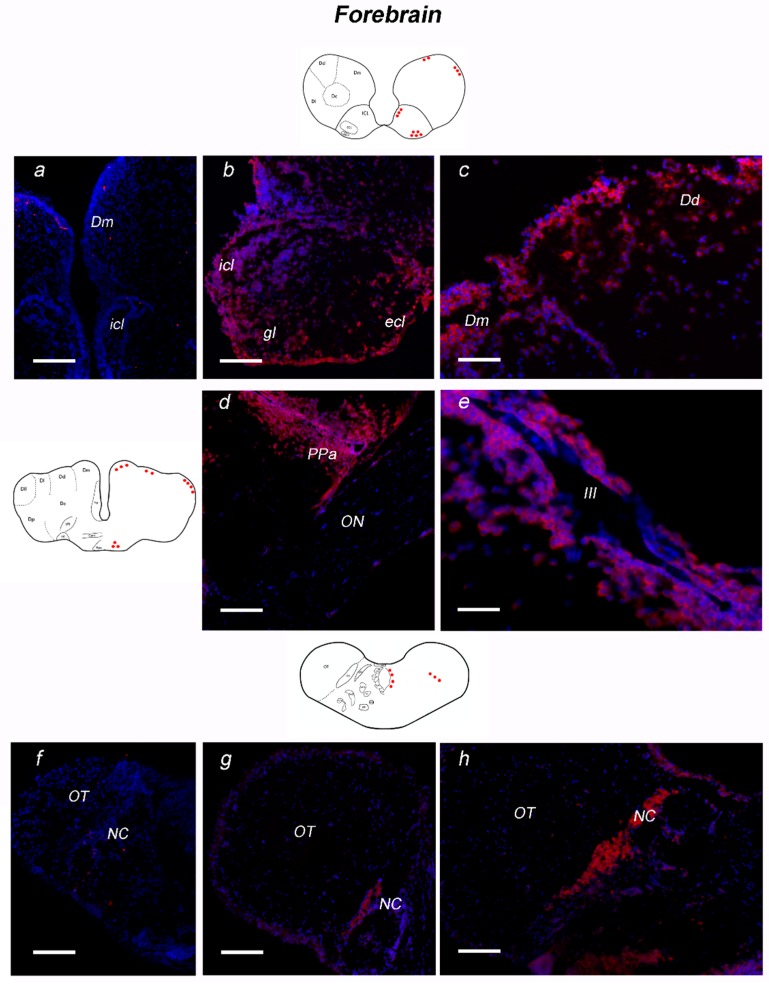
Expression of neurotrophin-6 (NT-6) mRNA in transverse section of forebrain of adult *N. furzeri.* On the left side, schematic drawings of *N. furzeri* brain, with red dots indicating NT-6 mRNA distribution over the different brain areas. Forebrain: (**a**) sense probe staining in the Dm and OB; (**b**) positive cells in icl, ecl and gl of olfactory bulbs; (**c**) positive neurons in the dorso-medial and dorso-dorsal zone of dorsal telencephalon; (**d**) numerous and intensely stained neurons in the anterior part of preoptic area; (**e**) intensely stained neurons scattered along the third ventricle; (**f**) sense probe staining in the NC and anterior part of OT; (**g**) overview of pretectal area and anterior part of optic tectum, with strong positive labeling in the neurons of NC; and (**h**) high magnification of positive neurons of NC. Scale bars: (**a**–**c**,**f**) 50 μm; (**d**) 25 μm; and (**e**) 100 μm. Abbreviations: Dd, dorsal zone of dorsal telencephalon; Dm, medial zone of dorsal telencephalon; ecl, external cellular layer; gl, glomerular layer; icl, internal cellular layer; NC, cortical nucleus; OB, olphactory bulb; ON, optic nerve; OT, optic tectum; PPa, anterior preoptic area; III, third ventricle.

In the olfactory bulb, numerous moderately labeled cells were found in the internal and external cellular layers, as well as in the glomerular layer (Figure 5a). In the dorsal telencephalon, the expression pattern of NT-6 mRNA was characterized by weak labeling in few scattered neurons of the central nucleus, whereas intense signal probe was observed in dorso-dorsal, medial (Figure 5b) and lateral nuclei. In the ventral telencephalon, NT-6 mRNA weakly labeled few neurons of dorsal and lateral nuclei. In the preoptic area, NT-6 mRNA expression was detected in the anterior (Figure 5c), parvo- and magnocellular nuclei, and in the suprachiasmatic nucleus. Intense staining was seen in neurons along third ventricle (Figure 5d). In the pretectal area, strong labeling was observed in numerous neurons of cortical nucleus (Figure 5e,f), as well as in neurons of parvocellular superficial pretectal nucleus. Intense staining was observed in few neurons of supraglomerular nucleus. NT-6 mRNA was observed in several weakly positive neurons of dorsal hypothalamus.

##### Midbrain

**Figure 6:**
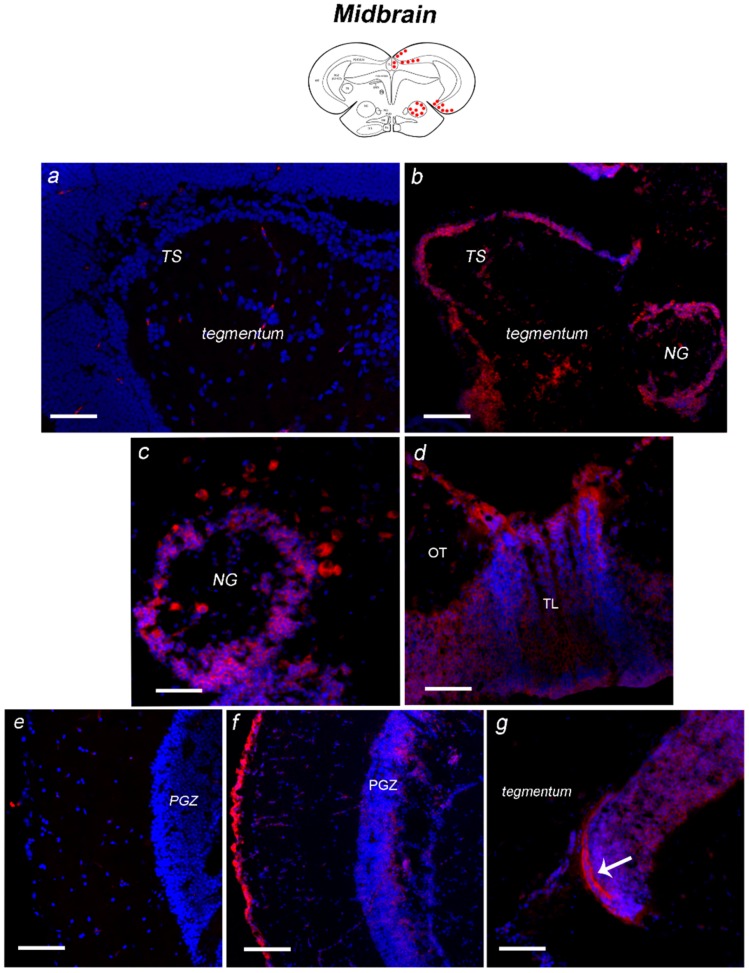
Expression of NT-6 mRNA in transverse section of caudal diencephalon/midbrain of adult *N. furzeri.* On the top, schematic drawings of *N. furzeri* brain, with red dots indicating NT-6 mRNA distribution over the different brain areas. Caudal diencephalon/midbrain: (**a**) sense probe staining in the anterior midbrain tegmentum; (**b**) high magnification of sense probe staining in the NG; (**c**) overview of tegmentum, with probe signal in neurons of TS and NG; (**d**) high magnification of positive neurons, scattered along the margin of NG; (**e**) probe signal in the dorsal and ventral part of TL and along the margin with OT; (**f**) few positive cells of PGZ in the anterior part of OT; and (**g**) numerous positive cells in the most caudal part of OT and intense staining at the margin between OT and the midbrain tegmentum (arrow). Scale bars: (**a**,**c**) 50 μm; and (**b**,**d**–**g**) 25 μm. Abbreviations: NG, glomerular nucleus; OT, optic tectum; PGZ, periventricular grey zone; TL, longitudinal tori; TS, semicircular tori.

**Figure 7:**
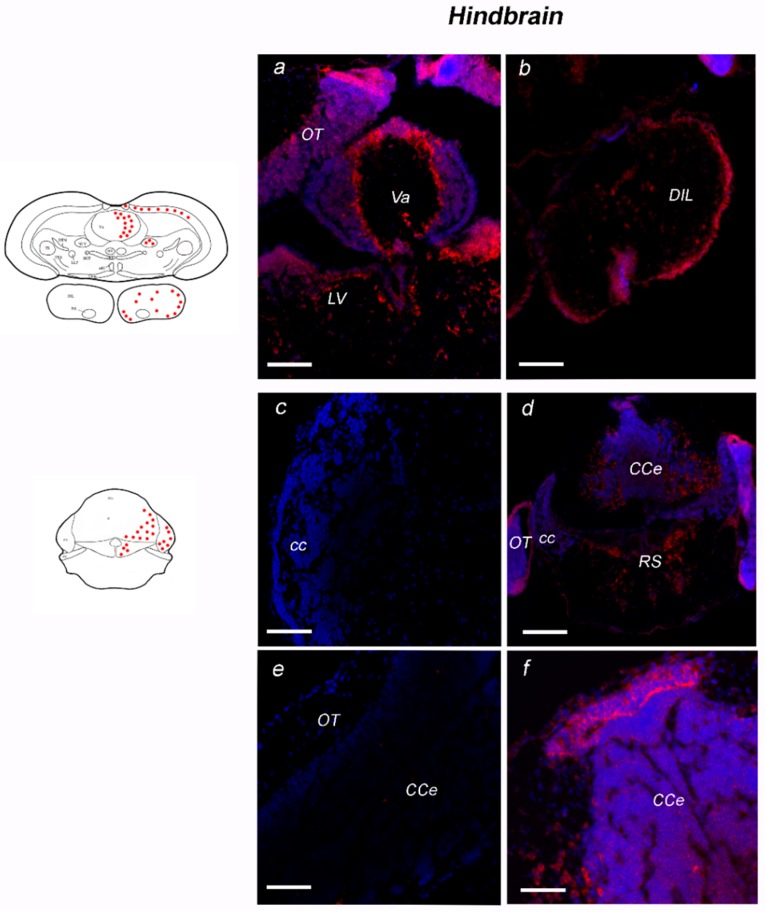
Expression of NT-6 mRNA in transverse section of hindbrain of adult *N. furzeri.* On the left side, schematic drawings of *N. furzeri* brain, with red dots indicating NT-6 mRNA distribution over the different brain areas. (**a**) Intense staining in the cells of caudal part of PGZ, and along the margin between OT and TL. Positivity in neurons of Purkinje layer of Va and in neurons of LV. (**b**) Positive neurons widespread over the central nucleus and dorsal hypothalamus of the most caudal part of DIL. (**c**) Sense probe staining in the cerebellar crista. (**d**) Intense staining in neurons of Purkinje, in neurons of RS. (**e**) Sense probe staining in the OT and CCe. (**f**) Intense staining labeling in the most upper portion of CCe (arrow) and in neurons of Purkinje layer of CCe. Scale bars: (**a**,**b**,**d**) 50 μm; and (**c**,**e**,**f**) 100 μm. Abbreviations: CCe, corpus of cerebellum; cc, cerebellar crista, DIL, diffuse inferior lobe of hypothalamus; LV, nucleus of lateral valvula; OT, optic tectum; PGZ, periventricular grey zone; RS, superior reticular formation; TL, longitudinal tori; Va, valvula of cerebellum.

The sense probe staining is shown in Figure 6a,b. Between forebrain and midbrain, moderate labeling was observed in neurons of the anterior glomerular nucleus, in neurons bordering the margins of the glomerular nucleus and in few large neurons in its inner part (Figure 6c,d). In the longitudinal tori, NT-6 mRNA was intensely expressed in numerous positive neurons located mainly in the most ventral part (Figure 6e), and along the margin with the optic tectum (Figure 6f). In the optic tectum, positive neurons were observed in the periventricular grey zone (Figure 6g and Figure 7a). However, NT-6 mRNA expressing neurons were few in the most rostral part of the periventricular grey zone (Figure 6f) while became more numerous caudally (Figure 6g and Figure 7a). Furthermore, the neurons lining the margin between the optic tectum and tegmentum were intensely labeled (Figure 6g). In the tegmentum, a positive signal was detected in neurons of Layers 1, 3 and 4 of semicircular tori (TS-1, TS-3, and TS-4) (Figure 6c).

##### Hindbrain

Strong labeling was observed in the most rostral region of cerebellum, with scattered neurons largely diffused in the lateral nucleus of cerebellar valvula (Figure 7a). In the most caudal part of the inferior lobe of hypothalamus, probe signal was seen in numerous small neurons of the diffuse nucleus and in large neurons of the central nucleus (Figure 7b).

**Figure 8:**
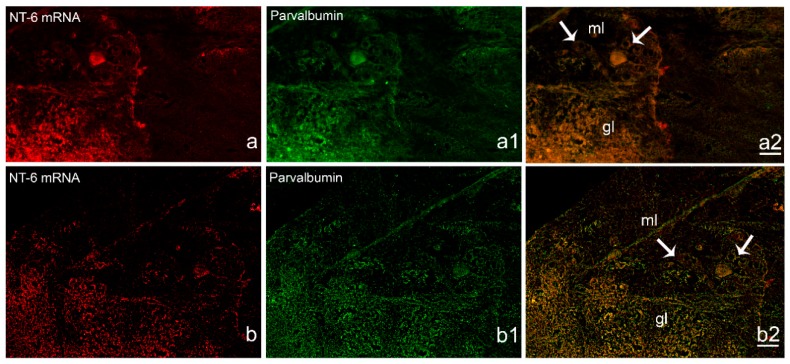
Immunohistochemical characterization of NT-6 mRNA expression in Purkinje cells in sagittal section of cerebellum. (**a**,**a1**,**a2**) single images and merge of NT-6 mRNA and Parvalbumin positive cells, showing co-staining in the gl and in Purkinje cells (arrow). (**b**,**b1**,**b2**) higher magnification of single images and merge of NT-6 mRNA and Parvalbumin positive cells showing co-staining in the gl and in Purkinje cells (arrow). Scale bar: a = 50 μm; b = 25 μm. Abbreviation: gl, granular layer; ml, molecular layer.

NT-6 mRNA was moderately localized in neurons of Purkinje layer of the lateral region of the cerebellar valvula (Figure 7a). Positive neurons in the Purkinje layer were also observed in the ventro-lateral and ventro-ventral subdivisions of cerebellar body (Figure 7c,d). The positive neurons were identified as Purkinje cells by double labeling with Parvalbumin (Figure 8a–b_2_). Strong labeling was seen also in elongated cells of the dorsal cerebellar subdivision (Figure 7c,d). Few positive neurons were labeled in the cerebellar crista (Figure 7c). In medulla oblongata, the expression pattern was seen in scattered neurons of octavolateral area, and in neurons of superior (Figure 7c) and intermediate reticular formation. Sense probe staining is shown in Figure 7e,f.

## 4. Discussion

The accumulated evidence documents an age-associated dysregulation of neurotrophins in the brain of mammals [38,39]. This is the first study reporting both the age-related expression of a neurotrophin in a non-mammalian vertebrate, and a comprehensive description of NT-6 mRNA in the adult brain of a fish species.

NT-6 has been identified in very few fish species: *Xiphophorus maculatus* [21], *Danio rerio* (zebrafish) [22], and *Cyprinus carpio* [23]. Phylogenetic analysis on neurotrophins, carried out on mature amino acid sequences [40,41], supports the hypothesis that during chordate/vertebrate lineage two rounds of duplication events of an ancestral neurotrophin gene occurred. Studies have shown that the genomic organization and transcript structure of NGF and NT-6 in the teleost zebrafish share a high similarity with the mouse NGF [24] and suggest that teleost NT-6 has evolved from a common ancestor after a single “fish specific” duplication of NGF [40,41]. Our phylogenetic studies on nucleotides sequences further confirm the hypothesis of NT-6 originated from NGF/NT-3 ancestors. However, the role of NT-6 needs to be clarified in teleosts fish: despite the degree of functional overlapping among the different neurotrophins, individual neurotrophins display a specific activity [42,43].

According to the few data available in literature, we hypothesize that this fish-specific neurotrophin might have a peculiar function among different fish species. Indeed, NT-6 persists in the brain of *N. furzeri* and the closest relative *Xiphophorus* [21] beyond early stages, whereas in zebrafish its expression is strictly linked to the embryonic stages [25]. The observation that NT-6 mRNA expression displayed comparable levels in the brain of young and old animals suggests us that, in our model species, this molecule plays a role in brain development as well as in its maintenance in adults. However, we cannot exclude that the expression levels, despite appearing very similar, can derive from a modulation of the NT-6 neuronal synthesis. For instance, it could be possible that few neurons express high levels of NT-6, or at the same time, that a high number of neurons express low levels of NT-6, in an age-dependent manner. Most interestingly, the synthesis of NT-6 in the aged brain could be a consequence of microglia activation, as compensatory mechanism of physiological aging process [44]. Further experiments are necessary to test these hypotheses and thus to better understand the role of neurotrophins in aging process. This is the first time we explored the age regulation of a neurotrophin in the brain of *N. furzeri*, while our previous studies had been addressed to investigate the morphological distribution of neurotrophins and their receptors in the adult brain.

Herein, we also provide a complete neuroanatomical description of NT-6 mRNA in the brain of *N. furzeri*. In this respect, we employed two different digoxigenin modified probes: an LNA probe and a riboprobe. LNA probes are generally used to detect short DNA oligonucleotides for microRNA and mRNA detection [30,45,46,47,48]. LNA containing DNA probes have been previously employed for in situ hybridization detection of mRNAs [45,46,47,48] in whole mount embryos of chicken and on tissue sections in *N. furzeri* [2]. The LNA probes revealed an enhanced hybridization efficiency, hybridization specificity and duplex stability [49]. Remarkably, our in situ experiments showed an overlapping neuroanatomical distribution for both probes.

Briefly, our results demonstrate that NT-6 mRNA is expressed in the forebrain (dorsal and ventral telencephalon, and in several diencephalic nuclei), in the midbrain (optic tectum, longitudinal tori, semicircular tori), and in the hindbrain (valvula and body of cerebellum, reticular formation and octavolateral area of medulla oblongata). NT-6 mRNA has been documented during the developing stages of *Xiphophorus* [21] and zebrafish [25], respectively, expressed in the valvula cerebelli and optic vescicle. In adult *Xiphophorus*, although NT-6 is expressed, a neuroanatomical description has not been reported yet [21]. Overall, our findings document a wide NT-6 mRNA localization throughout the whole brain of *N. furzeri*. In addition, other neurotrophins (NGF, BDNF and NT-4), at either mRNAs or protein level, were observed in the adult brain of *N. furzeri* [48,49,50]. Remarkably, NT-6 mRNA is expressed in mature neurons, similar to other neurotrophins, such as BDNF, NGF, and NT-4 [50,51,52], which have been already documented in the brain of this model species. Most interestingly, neurotrophins display a peculiar neuronal expression also in zebrafish and the European eel [35,53,54,55]. These observations reinforce the hypothesis that the expression of neurotrophins in teleost fish is primarily linked to mature neurons. In adult mammalian brain, the neuronal mRNA levels of NGF and BDNF are tightly regulated by neural activity and influence the modulation of several key events such as synthesis, metabolism and release of neurotransmitters, postsynaptic ion channel fluxes, neuronal firing rates as well as long-term synaptic potentiation of neurons [56]. In this context, further studies are mandatory to explore the evolutionary conserved neuronal role of neurotrophins in the brain of fish species.

In conclusion, our findings document: (1) the identification and molecular characterization of NT-6 coding sequence of *N. furzeri* (NfuNT-6) and the nucleotide degree of conservation in *Xiphophorus*, *D. rerio* and mammalian NGF and BDNF; (2) the efficiency of using a sensitive LNA probe to detect NT-6 mRNA; (3) the unchanged expression levels of NT-6 in the brain of young and old animals; and (4) the expression of NT-6 mRNA in mature neurons of forebrain, midbrain and hindbrain in *N. furzeri*. These results provide a basis for future research on evolutionary function of neurotrophins, which are currently perceived as one of the primary factors underlying the complexity of vertebrate nervous systems. Therefore, their involvement in higher brain functions and aging is undoubtedly a relevant topic.

Further experimental work is noticeably needed both to characterize more in-depth NT-6 in this model and to confirm its importance in brain development and architecture. In addition, functional studies are required to explore and feature the potential role played by NT-6 in aging.

## Figures and Tables

**Table 1:** LNA probe and mismatch probe.

Target mRNA	5′-Mod	Synthesis Sequence (5′–3′)	3′-Mod
NT-6	DIG	TTGTCTCCTGCTGTCCTGCTCTG	DIG
* mut NT-6	DIG	TTGTCTC**TC**GCTG**CT**CTGCT**TC**G	DIG

* Mutations are shown bolded.
